# A multicenter, retrospective epidemiologic survey of the clinical features and management of bone metastatic disease in China

**DOI:** 10.1186/s40880-016-0102-6

**Published:** 2016-04-25

**Authors:** Yunpeng Yang, Yuxiang Ma, Jin Sheng, Yan Huang, Yuanyuan Zhao, Wenfeng Fang, Shaodong Hong, Ying Tian, Cong Xue, Li Zhang

**Affiliations:** Department of Medical Oncology, Sun Yat-sen University Cancer Center, State Key Laboratory of Oncology in South China, Collaborative Innovation Center for Cancer Medicine, 651 Dongfeng Road East, Guangzhou, 510060 Guangdong P. R. China

**Keywords:** Bisphosphonates, Bone metastases, Skeleton-related events, Chinese cancer patients

## Abstract

**Background:**

Bone metastases are common in patients with advanced cancer. Bisphosphonates (BPs) could prevent or delay the development of skeleton-related events (SREs). The present study aimed to identify the clinical features of and treatment strategies for Chinese patients with bone metastases.

**Methods:**

Consecutive cancer patients who had bone metastases and received BP treatment were enrolled. A questionnaire was developed to collect the patients’ clinical data, as well as information on the diagnosis and management of bone metastases. Physicians’ awareness of the guidelines and knowledge of the application of BP were also assessed.

**Results:**

A total of 3223 patients with lung cancer (36.5%), breast cancer (30.9%), prostate cancer (8.5%), and gastrointestinal cancer (5.7%) were included in this study. The sites of bone metastases were the thoracic spine (56.0 %), lumbar spine (47.1%), ribs (32.6%), and pelvis (23.2%). The SRE frequency was the highest in patients with multiple myeloma (36.6%), followed by those with lung cancer (25.9%), breast cancer (20.2%), prostate cancer (18.2%), and gastrointestinal cancer (17.3%). Irradiation to the bone was the most frequent SRE (58% in lung cancer patients, 45% in breast cancer patients, and 48% in prostate cancer patients). Our survey also showed that 45.5% of patients received BP within 3 months after their diagnosis of bone metastases, whereas the remaining 54.5% of patients did not receive BP treatment until at least 3 months after their diagnosis of bone metastases. The SRE frequency in the former group was significantly lower than that in the latter group (4.0% vs. 42.3%, *P* < 0.05). In patients with more than 6 months of continuous BP treatment, the mean time to the first SRE was significantly longer than that in patients with less than 6 months of continuous BP treatment (7.2 vs. 3.4 months, *P* < 0.05). In addition, 12.2% of the physicians were not aware of the efficacy of BP in preventing and delaying SRE. Only half (52.3%) of the physicians agreed that the BP treatment should persist for at least 6 months unless it was intolerable.

**Conclusions:**

Our study suggested that prompt and persistent BP treatment was associated with a reduced risk of SREs. However, our survey also revealed that the proper application of BP was not as common as expected in China.

**Electronic supplementary material:**

The online version of this article (doi:10.1186/s40880-016-0102-6) contains supplementary material, which is available to authorized users.

## Background

Advanced cancer is often associated with metastasis, and the bone is a common metastatic site [1, 2]. Bone metastases are frequently associated with certain types of cancer. For example, bone metastases occur in 70% of breast or prostate cancer patients and in 40% of lung cancer patients [2]. Bone metastases usually result in reduced survival of patients with advanced cancer [3, 4]. Furthermore, bone metastases also substantially affect the patient’s quality of life (QoL) due to skeleton-related events (SREs), such as pathologic fractures, the need for bone radiotherapy and surgery, spinal cord compression, and hypercalcemia.

The prevention and delay of SREs and improvements in the patient’s QoL are the primary goals for the clinical management of bone metastases. Due to the diversity and complexity of the skeletal complications, the treatment of bone metastases often involves multidisciplinary approaches. The use of bisphosphonates (BPs) has played an essential role in the management of bone metastases. As analogues of endogenous pyrophosphate, which regulates mineral turnover in the bone, BPs bind to the hydroxyapatite crystals in the bone and inhibit bone resorption. Previous studies have shown that BPs can prevent and delay SREs, thus reducing the risk of skeletal complications in patients with bone metastases [5–8]. In particular, the third-generation BPs, which are superior to first- or second-generation BPs, have shown significantly improved efficacy and have been widely used for the treatment of bone metastases [9–12]. Therefore, the guidelines have recommended the application of BPs as a standard therapy for bone metastases [3, 13–15].

To improve the management of bone metastases in China, the Chinese Anti-Cancer Association and the Professional Committee of Cancer Rehabilitation and Palliative Care together released the Chinese Expert Consensus Statement on the clinical diagnosis and treatment of bone metastases in 2007 [16], with an updated version released in 2014 [17]. As diagnostic and therapeutic guidelines for Chinese clinicians, the Expert Consensus Statement presented specific recommendations for diagnosing and managing bone metastases. In the statement, each treatment option for bone metastases, including analgesia, radiotherapy, surgery, and supportive treatment, was reviewed in detail. In particular, the statement fully recognized the effectiveness and long-term safety of BPs and recommended that BP treatment should start as soon as bone metastases is diagnosed and last longer than 6 months if the patients tolerate the treatment. However, there are very few reports on the frequency of BP application and its associated clinical outcomes in China.

To identify the characteristics of bone metastasis management in China and the underlying factors that affect the clinical outcomes, we conducted a retrospective epidemiologic survey in more than 50 hospitals throughout the country. By documenting the types of cancer, sites of bone metastases, SRE occurrence, diagnostic methods, and treatment of bone metastases, we expected to provide a database on the local patterns of bone metastasis management and provide evidence for the effect of BPs on bone metastases in Chinese patients.

## Patients and methods

### Patients

The participants in this study were consecutively recruited from 53 hospitals in 30 Provinces in China between January 2008 and August 2009. The patient inclusion criteria were as follows: diagnosis of cancer, presence of bone metastases, and receiving BP treatment for bone metastases at the time of enrollment. The protocol was approved by the institutional review boards at each participating institution. All patients provided written informed consent.

### Survey design

In the present study, a questionnaire (Additional file 1) was developed to collect the information. The questionnaires were completed by physicians with the consent of each patient. Using this questionnaire, we collected the patients’ basic clinical and demographic data, including age, gender, Eastern Cooperative Oncology Group performance status (ECOG PS) score, primary tumor type and stage, date of initial cancer diagnosis, and treatment for the primary tumor. The questionnaire also collected information on the diagnosis and management of bone metastases, including the date of the initial bone metastasis diagnosis, diagnostic tests for bone metastases, sites of bone metastases, treatments for bone metastases, number and types of SREs before and after the initial bone metastasis diagnosis, date of first SRE, date of first BP treatment, duration of BP treatment, types and dosage of BPs, and adverse effects during BP treatment. The physicians were asked to provide anonymous answers regarding their awareness of the Chinese Expert Consensus Statement on the clinical diagnosis and treatment of malignant tumor bone metastasis and bone-related diseases, as well as their knowledge on the use of BPs for the management of bone metastases.

### Statistical analyses

The baseline characteristics of the patients were determined using descriptive statistics. The quantitative variables were analyzed with an independent-sample *t* test. Pearson’s χ^2^ test and Fisher’s exact test were used to examine the differences in the distributions of the categorical variables as appropriate. Statistical significance was defined as a value of *P* < 0.05. SPSS for Windows software, version 19.0 (IBM, Chicago, IL, USA) was used for all data analyses. All significance levels refer to two-sided tests.

## Results

### Patient characteristics

Between January 2008 and August 2009, 3223 eligible patients were enrolled into the study. The patients’ demographic and clinical characteristics are shown in Table 1. The most common cancer types were lung (36.5%), breast (30.9%), prostate (8.5%), and gastrointestinal cancers (5.7%).

**Table 1:** Demographic and clinical characteristics of the 3223 patients with bone metastases

Characteristic	Number of patients	Percent (%)
Age (years)		
<50	935	29.0
50–70	1673	51.9
>70	615	19.1
Gender		
Male	1515	47.0
Female	1708	53.0
ECOG PS		
0	617	19.1
1	968	30.0
≥2	1638	50.8
Cancer type		
Lung cancer	1178	36.5
Breast cancer	996	30.9
Prostate cancer	274	8.5
Gastrointestinal cancer	185	5.7
Multiple myeloma	112	3.5
Others	478	14.8
Metastatic sites other than the bone		
Yes	1882	58.4
No	1341	41.6

*ECOG PS* Eastern Cooperative Oncology Group performance status

### Diagnosis of bone metastases

In this study, the diagnostic methods were documented for 2979 patients. To screen for bone metastases, 481 (16.1%) patients received a radionuclide bone scan, 1793 (60.2%) received a radionuclide bone scan with further imaging tests (X-ray radiography, computed tomography, or magnetic resonance imaging), and 688 (23.1%) received an imaging examination without a radionuclide bone scan. Notably, there were 17 patients (0.6%) whose diagnosis was based solely on the symptoms or laboratory results, without either imaging or radionuclide evidence.

### Sites of bone metastases

Typically, multiple sites were affected by bone metastases. We collected information on the sites of bone metastases for 3039 patients, and 2578 (84.8%) of them had multiple bone metastases. The information on the bone metastatic sites for 184 patients was missing. The most common bone metastatic site was the thoracic spine (*n* = 1702, 56.0%), followed by the lumbar spine (*n* = 1431, 47.1%), ribs (*n* = 990, 32.6%), pelvis (*n* = 705, 23.2%), femur (*n* = 552, 18.2%), and other sites (*n* = 469, 15.4%), such as the humerus and skull.

### Occurrence and types of SREs

**Fig. 1:**
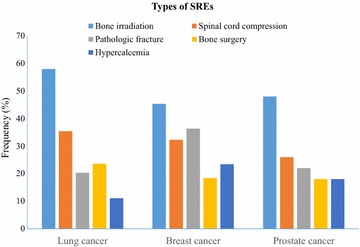
Types of skeleton-related events (SREs) in patients with lung, breast, or prostate cancer. The frequency of bone irradiation was the highest in these patients

A total of 745 (23.1%) patents had at least one SRE. The SRE frequency was the highest in patients with multiple myeloma (41/112, 36.6%), followed by those with lung cancer (305/1178, 25.9%), breast cancer (201/996, 20.2%), prostate cancer (50/274, 18.2%), and gastrointestinal cancer (32/185, 17.3%). The types of SREs in the three most common cancers (lung, breast, and prostate cancers) are shown in Fig. 1. The frequency of bone irradiation was the highest (58.0% for lung cancer patients, 45.3% for breast cancer patients, and 48.0% for prostate cancer patients) compared with other types of SREs. In patients with lung cancer or prostate cancer, the frequency of spinal cord compression (35.4% for lung cancer patients and 26.0% for prostate cancer patients) was second to that of radiotherapy. In patients with breast cancer, however, the frequency of pathologic fracture (36.3%) was second to that of bone irradiation.

### Application of BPs and its association with SREs

According to the inclusion criteria of the survey, all of the enrolled patients received BP therapy. The majority of the patients (80.1%, *n* = 2583) were treated with third-generation BPs, 19.5% (*n* = 629) received second-generation BPs, and only 0.3% (*n* = 11) received first-generation BPs.

The time at which BP therapy was initiated was documented for 2994 patients, of which 1363 (45.5%) patients received BPs at the same time or within 3 months after their diagnosis of bone metastases, whereas 1631 (54.5%) patients did not start BP treatment until at least 3 months after their diagnosis of bone metastases. Notably, the SRE frequency in the former group was significantly lower than that in the latter group (4.0% vs. 42.3%, *P* < 0.05).

**Fig. 2:**
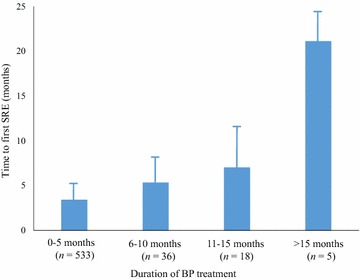
Relationship between the duration of bisphosphonate (BP) treatment and the time to the first occurrence of SREs. *Column*, average value; *bar*, standard deviation

Among the 745 patients who had experienced at least one SRE, the duration of the BP treatment was known for 592 patients. The overall average length of BP treatment was 2.71 months. To investigate the efficacy of BPs in delaying SREs, we examined the relationship between the duration of BP treatment and the time to the first SRE. As shown in Fig. 2, the interval between the diagnosis of bone metastases and the first SRE increased as the BP treatment duration increased. In patients whose BP treatment persisted for 6 months or longer, the mean time to the first SRE was significantly longer than that of the patients who had less than 6 months of BP treatment (7.2 vs. 3.4 months, *P* < 0.05).

### Physicians’ awareness of the consensus and knowledge on the use of BPs

A total of 740 physicians responded to the questions in our questionnaire. Approximately 10.3% of the physicians claimed they did not use the Chinese Expert Consensus Statement as their clinical guideline for the treatment of bone metastases. Additionally, 12.2% of the physicians were not aware of the efficacy of BPs in preventing and delaying SREs in patients with bone metastases. Notably, only half of the physicians (52.3%) agreed that the BP treatment should persist for at least 6 months unless the treatment was intolerable.

## Discussion

In this survey, we found that the most common cancer type with bone metastases was lung cancer, the most frequently affected bone metastatic sites were the axial skeletons, and irradiation to the bone was the most frequent SRE in Chinese patients. We also found that the delayed initiation and non-persistent use of BP treatment were common in China, which significantly increased the risk of SREs. Furthermore, Chinese clinicians’ awareness of the guidelines and knowledge on bone metastasis management were not as good as expected, which might lead to inappropriate applications of BPs.

Although it has been reported that the bone metastasis rate in breast or prostate cancer patients was higher than that in lung cancer patients [3, 18, 19], the most common cancer type with bone metastases in this survey was lung cancer. This result can be explained by that the incidence of lung cancer is higher than those of breast and prostate cancers both worldwide [1] and in China [20]. Consistent with previous studies **[**2, 15], the axial skeletons were frequently affected by bone metastases in our survey. SREs are more likely to occur in the axial skeletons than in the peripheral bones and are associated with the loss of mobility and social functioning, a decrease in the patients’ QoL, and a substantial increase in medical costs. Furthermore, SREs can increase the mortality of patients with bone metastases. According to a large cohort study in Denmark, the 5-year survival rate was 8.3% for breast cancer patients suffering from bone metastases without SREs; in contrast, the rate was only 2.5% for those with both bone metastases and SREs [21]. A similar study of 9746 prostate cancer patients with bone metastases conducted in the United States also found that the mortality was significantly higher in those with both bone metastases and SREs than in those with bone metastases, but no SREs [22]. These findings highlight the importance of managing bone metastases from the time of diagnosis to reduce the risk of SREs.

In our study, 76.3% of the patients received a radionuclide bone scan to screen for bone metastases, and 16.1% of the patients were diagnosed with bone metastases solely based on the radionuclide bone scan results, without confirmation by additional imaging techniques. Although the bone scan is a relatively sensitive and inexpensive examination that evaluates the entire skeleton, its false positive rate is very high, ranging from 30% to 40% [23, 24]. Thus, the guidelines recommend that positive findings in the bone scan should be confirmed by X-ray radiography, magnetic resonance imaging (MRI), computed tomography (CT) scan, or positron emission tomography-computed tomography (PET-CT) [13, 15, 16]. Furthermore, the diagnosis for 17 patients (0.6%) in our study was based solely on their symptoms or laboratory results, without bone scan or imaging evidence. These findings suggest that the diagnosis of bone metastases in China should be improved in the future.

Several large prospective clinical trials have demonstrated that BPs effectively prevent and delay SREs in cancer patients with bone metastases [8, 25–27]. However, the inappropriate application of BPs would substantially compromise their effectiveness. A large retrospective study of 2212 breast cancer patients with bone metastases found that the risk of SREs was as high as 47.02% in the patients with a delayed initiation of BP treatment (more than 2 months after the diagnosis of bone metastases) [28]. Similarly, we showed that the risk of SREs was 4.0% in patients with early BP treatment, whereas the risk was increased to 42.3% in patients with delayed BP treatment. Thus, the European Society for Medical Oncology (ESMO) [3], National Comprehensive Cancer Network (NCCN) [15], and Chinese guidelines [16] all recommend that BP treatment should be initiated once malignant bone metastases was confirmed. Moreover, the persistent use of BPs is also very important to prevent and delay SREs. For example, studies showed that persistent, monthly use of BPs for more than 18 months was associated with a 50% lower risk of SREs compared with persistent, monthly use of BPs for less than 3 months in breast cancer patients with bone metastases [28]. In addition, even for patients with solid tumors who already received BP treatment for at least 2 years, the persistent use of BPs for an additional 18 months was associated with a lower SRE risk (hazard ratio = 0.42, *P* = 0.01) [29]. Our survey also showed that the persistent use of BPs for more than 6 months could significantly prolong the time to the first SRE compared with the use of BPs for less than 6 months. Therefore, BP treatment should be continued until it is no longer tolerated by the patient or the occurrence of substantial decline in the patient’s general performance status [13–16].

Unfortunately, our study showed that the use of BPs in China was unsatisfactory. Our survey indicated that more than half of the patients did not receive BP treatment as soon as bone metastases were diagnosed. Furthermore, the majority of the patients received less than 6 months of BP treatment, and only 10% of patients received more than 6 months of persistent BP treatment. It appeared that the delayed initiation of the treatment and its poor persistency could be attributed, at least partially, to clinician-associated factors. As indicated in our survey, up to 12.2% of the physicians were not aware of the efficacy of BPs in preventing SREs, and nearly half of the physicians did not support the long-term use of BPs. Undoubtedly, significant efforts are required to improve the clinicians’ awareness of the guidelines and knowledge about bone metastasis management.

The limitations of this study should be considered in the interpretation of the findings. First, this is a retrospective study, and thus the observed associations among variables might not be causal. Second, because this study only included the bone metastasis patients who received BPs, the frequency of BP application in the entire bone metastasis population remains to be determined.

## Conclusions

In summary, our study described the characteristics of bone metastases and its management in Chinese cancer patients. The results suggested that prompt and persistent BP treatment is associated with a reduced risk of SREs. However, our survey also revealed that the proper application of BPs was not as common as expected in China. There is an urgent demand to improve clinicians’ awareness of applying BPs for bone metastasis management.

## Additional file

10.1186/s40880-016-0102-6 Questionnaire for survey of the clinical features and management of bone metastatic disease in China.
