# Supplementary material for: A multicenter, retrospective epidemiologic survey of the clinical features and management of bone metastatic disease in China
Source: Chin J Cancer. 2016 Apr 25;35:40. doi: 10.1186/s40880-016-0102-6 (PMC4845386; doi:10.1186/s40880-016-0102-6)
Supplement: Supplementary file 1 — 10.1186/s40880-016-0102-6 Questionnaire for survey of the clinical features and management of bone metastatic disease in China. [file 40880_2016_102_MOESM1_ESM.docx]

Supplementary Questionnaire

**Hospital serial No.:** __________

**Investigation on the status quo of diagnosis and treatment for bone metastasis —— patient first registration table**

**Part I Patient’s basic information:**

| Initials |  | **Date of birth** | DDMMYYYY |
| --- | --- | --- | --- |
| Gender | Male Female | City |  |
| Doctor in charge |  | Hospital department |  |

**Part II Patient’s diagnosis information:**

| Primary tumor |  | Tumor stage |  |
| --- | --- | --- | --- |
| Time of definite diagnosis | DDMMYYYY | TNM stage |  |
| Sites of bone metastasis (please check) | Thoracic vertebrae | Lumbar vertebrae | Pelvis |
|  | Femoral | Ribs | Others____________ |
| Time of definite diagnosis of bone metastasis | DDMMYYYY | | |
| Diagnosis methods (please choose) | Physical signs | ECT | X-ray |
|  | CT | MRI | Bone density |
|  | NTX-N-terminal peptide | ICTP-I type collagen c-peptide | Akaline phosphatase |
| Other metastatic sites (please choose) | Lung | Liver | Brain |
| Corresponding time of definite diagnosis | DDMMYYYY | DDMMYYYY | DDMMYYYY |

**Part III Relevant symptoms, degree, and occurrence frequency of bone metastasis before treatment:**

| Do the following skeleton-related events (SRE) occur when the diagnosis of bone metastasis is finalized? Yes No | | | |
| --- | --- | --- | --- |
| Bone pain | Yes No | Pain grade |  |
| Hypercalcemia | Yes No | Level |  |
| Pathologic fracture | Yes No | Occurrence frequency and time |  |
| Spinal compression caused by bone metastasis | Yes No | Occurrence frequency and time |  |
| Receiving surgery due to bone metastasis | Yes No | Occurrence frequency and time |  |
| Receiving radiotherapy due to bone metastasis | Yes No | Occurrence frequency and time |  |
| ECOG score |  | | |

**Part IV Relevant symptoms, degree, and occurrence frequency of bone metastasis after definite diagnosis of bone metastasis:**

| Do the following skeletal-related events (SRE) occur during the treatment after definite diagnosis of bone metastasis? Yes No | | | |
| --- | --- | --- | --- |
| Bone pain | Yes No | Pain grade |  |
| Hypercalcemia | Yes No | Level |  |
| Pathologic fracture | Yes No | Occurrence frequency and time |  |
| Spinal compression caused by bone metastasis | Yes No | Occurrence frequency and time |  |
| Receiving surgery due to bone metastasis | Yes No | Occurrence frequency and time |  |
| Receiving radiotherapy due to bone metastasis | Yes No | Occurrence frequency and time |  |
| ECOG score |  | | |

**Part V Patient’s SRE, bone metastasis, and diphosphonate treatment:**

| Time of definite diagnosis of bone metastasis in patients | Time of occurrence | DDMMYYYY |
| --- | --- | --- |
| Time of first occurrence of SRE in patients | Time of occurrence | DDMMYYYY |
| Duration of treating bone metastasis with diphosphonate used by patients | Time of occurrence | DDMMYYYY |

**Part VI Patient's medical history**

|  | Treatment of current primary diseases  Used before? | Treatment after bone metastasis  Used? | Main treatment regimen | Treatment of bone metastasis  Start and end date |
| --- | --- | --- | --- | --- |
| Diphosphonate treatment | Yes No | Yes No |  | (DDMMYYYY) to (DDMMYYYY) |
| Radiotherapy of bone metastasis | Yes No | Yes No |  | (DDMMYYYY) to (DDMMYYYY) |
| Nuclide treatment | Yes No | Yes No |  | (DDMMYYYY) to (DDMMYYYY) |
| Chemotherapy | Yes No | Yes No |  | (DDMMYYYY) to (DDMMYYYY) |
| Endocrine therapy | Yes No | Yes No |  | (DDMMYYYY) to (DDMMYYYY) |
| Surgery | Yes No | Yes No |  | (DDMMYYYY) to (DDMMYYYY) |
| Analgesic treatment | Yes No | Yes No |  | (DDMMYYYY) to (DDMMYYYY) |
| Others (please describe)  ______________ | Yes No | Yes No |  | (DDMMYYYY) to (DDMMYYYY) |
| Please complete the following part as for the specifics of treatment of bone metastasis | | | | |

**Part VII Treatment of bone metastasis with diphosphonate**

| Name of drug | Dosage | Treatment start and end date | Total duration |
| --- | --- | --- | --- |
| First-generation diphosphonate | mg | (DDMMYYYY) to (DDMMYYYY) | Month(s) |
| Second-generation diphosphonate | mg | (DDMMYYYY) to (DDMMYYYY) | Month(s) |
| Third-generation diphosphonate | mg | (DDMMYYYY) to (DDMMYYYY) | Month(s) |

**Part VIII Common circumstances of diphosphonate treatment**

| As long-term use is needed for most diphosphonate drugs, can the patient stick to long-term use: Yes No | | | |
| --- | --- | --- | --- |
| The reasons you think for which patients cannot stick to the long-term use of diphosphonate drugs? | | | |
| The drugs are too expense | The drugs cannot be used in outpatient departments | No significant efficacy |  |
| Drug withdrawal or dosage reduction? | Yes No |  |  |

**Part IX Doctor's understanding on Chinese experts' consensus on bone metastasis**

| Understand the Chinese experts' consensus on bone metastasis | Yes No |
| --- | --- |
| Use diphosphonate by referring to Chinese experts' consensus on bone metastasis in clinical work | Yes No |
| Diphosphonate can effectively prevent or delay the incidence of SRE caused by bone metastasis | Yes No |
| For patients who just start the administration of diphosphonate, the administration should persist until the patient's general conditions become significantly relieved or the patients cannot tolerate the symptoms. The duration should be at least 6 Months under general circumstances | Yes No |
